# Tracheal intubation in patients with Pierre Robin sequence: development, application, and clinical value based on a 3-dimensional printed simulator

**DOI:** 10.3389/fphys.2023.1292523

**Published:** 2024-02-01

**Authors:** Yu Mao, Lu Liu, John Zhong, Pei Qin, Rui Ma, Mingzhang Zuo, Li Zhang, Lifang Yang

**Affiliations:** ^1^ Department of Cardiovascular Surgery, Xijing Hospital, Air Force Medical University, Xi’an, China; ^2^ Department of Anesthesiology, Children's Hospital of Nanjing Medical University, Nanjing, China; ^3^ Department of Anesthesiology and Pain Management, University of Texas Southwestern Medical Center, Dallas, TX, United States; ^4^ Department of Anesthesiology, Xi’an Children Hospital, Xi’an, China; ^5^ Department of Anesthesia, Beijing Hospital, National Center of Gerontology, Institute of Geriatric Medicine, Chinese Academy of Medical Sciences, Beijing, China

**Keywords:** Pierre Robin sequence, tracheal intubation, 3-dimensional printing, simulator, difficult airway

## Abstract

**Background:** The main clinical manifestations of patients with Pierre Robin sequence (PRS) include micrognathia, the glossoptosis and dyspnoea. The difficulty of tracheal intubation (TI) in such patients is increased.

**Objective:** The purpose of the study was to evaluate the reliability and efficacy of the PRS simulator.

**Methods:** A PRS simulator was developed by using 3-dimensional (3D) printing technology under computer-aided design. A total of 12 anaesthesiologists each trained 5 times for TI on the PRS Training Simulator-1 and recorded the simulation time. After the training, they were randomly divided into three groups with a total of 12 nontrained anaesthesiologists, and the simulation was completed on PRS Simulator-2, 3 and 4. The simulation time was recorded, and the performance was evaluated by three chief anaesthesiologists. Then, all 24 anaesthesiologists completed the questionnaire.

**Results:** A PRS simulator developed by 3D printing was used to simulate the important aspects of TI. The average number of years worked was 6.3 ± 3.1 years, and 66.7% were female. The time for the 12 anaesthesiologists to complete the training gradually decreased (*p* < 0.01). Compared with the trained anaesthesiologists, the simulation time of TI in the nontrained anaesthesiologists was much longer (all *p* < 0.01). In addition, the simulation performance of the trained anaesthesiologists was relatively better (all *p* < 0.01).

**Conclusion:** The reliability and efficacy of the PRS simulator is herein preliminarily validated, and it has potential to become a teaching and training tool for anaesthesiologists.

## Introduction

Pierre Robin sequence (PRS) is a rare congenital defect caused by mandibular dysplasia in new-borns characterised by micrognathia, glossoptosis and airway obstruction (Robin, 1923). PRS, if not handled in time, will lead to a series of serious complications (Cole et al., 2008). The treatment of PRS generally involves postural therapy, continuous positive pressure ventilation, nasopharyngeal ventilation, orthodontic treatment and other nonsurgical treatments. When nonsurgical treatment is ineffective or difficult to maintain, surgical treatment is needed. In recent years, mandibular distraction osteogenesis (MDO) has become the main surgical method for PRS treatment and has achieved good therapeutic effects, replacing tracheotomy and tongue and labial adhesions (TLAs) (Gómez et al., 2018).

Successful airway assessment and management is important in pre-MDO anaesthesia. However, the unique anatomy of PRS undoubtedly increases the challenge of tracheal intubation (TI), which in turn can lead to fatal anaesthesia-related complications (Artime and Hagberg, 2015). It has been reported that one in every 22,000 patients undergoing anaesthesia develops serious airway-related complications (Cook et al., 2011). Among them, the major associated adverse outcomes include death, hypoxic brain injury, cardiopulmonary arrest, airway trauma, aspiration of stomach contents, pulmonary oedema, and dental damage (Cook et al., 2011).

In recent years, 3-dimensional (3D) printing has become an easy technology to use and versatile. It is a technology that is based on the 3D reconstruction data formed by medical imaging scans and printed layer by layer of the corresponding model exactly consistent with the reconstructed model (MacDonald and Wicker, 2016). This technology has been widely applied in orthopaedics, neurosurgery and orofacial surgery, but its use in anaesthesia has not yet been full-scale (Sinn et al., 2006; Suomalainen et al., 2015; Mulford et al., 2016). Previous studies have shown that the use of 3D printing to create a patient’s airway model can help anaesthesiologists more fully understand the morphology of the trachea, choose the appropriate catheter, and determine a more secure intubation approach in advance (Fiadjoe et al., 2015; Park et al., 2021; Shaylor et al., 2023). Furthermore, in real-world procedures, the intubation time has been shortened, the success rate of intubation has been increased, and complications caused by repeated intubation have mainly been avoided (Han et al., 2016).

Therefore, to enhance familiarity of anaesthesiologists’s handling difficult airway such as PRS and potentially lower complications caused by mishandling, we designed an innovative, highly realistic PRS simulator to guide anaesthesiologists to simulate and practice key parts of procedures. The purpose of this study was to evaluate the reliability and efficacy of the simulator for preprocedural evaluation and training in such patients.

## Methods

### Study design

**FIGURE 1:**
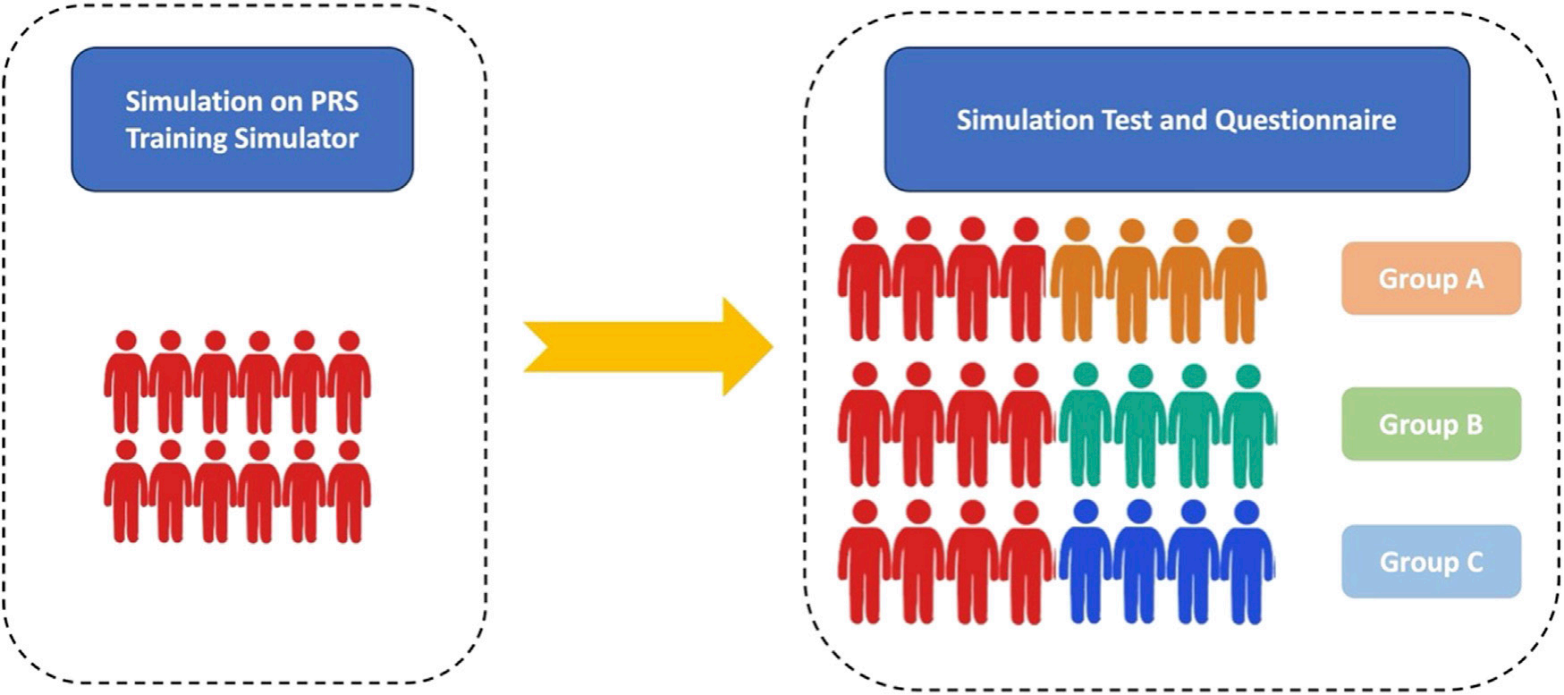
Flow chart. Red figures represent the trained anaesthesiologists. Orange, green and blue figures represent the nontrained anaesthesiologists in Groups A, B, and C, respectively.

Four patients with PRS who received MDO treatment in Nanjing Children’s Hospital and Xi’an Children’s Hospital in May 2023 were enrolled. Based on their computed tomography (CT) data, 3D printed models were built and printed by professional engineers under computer-aided design, which were named PRS Training Simulator (Patient #1) and PRS Testing Simulator-1 (Patient #2), 2 (Patient #3), and 3 (Patient #4). Twenty-four anaesthesiologists from Xi’an Children’s Hospital and Nanjing Children’s Hospital were randomly assigned to participate in this study and to confirm the effects of the simulator (Figure 1). PRS Testing Simulator-2,3 and 4 were used in Groups A, B and C, respectively. This study protocol was approved by the Ethics Committee of the two centres (202301005-1), and all patient data were desensitised.

### Development of PRS simulator for tracheal intubation

**FIGURE 2:**
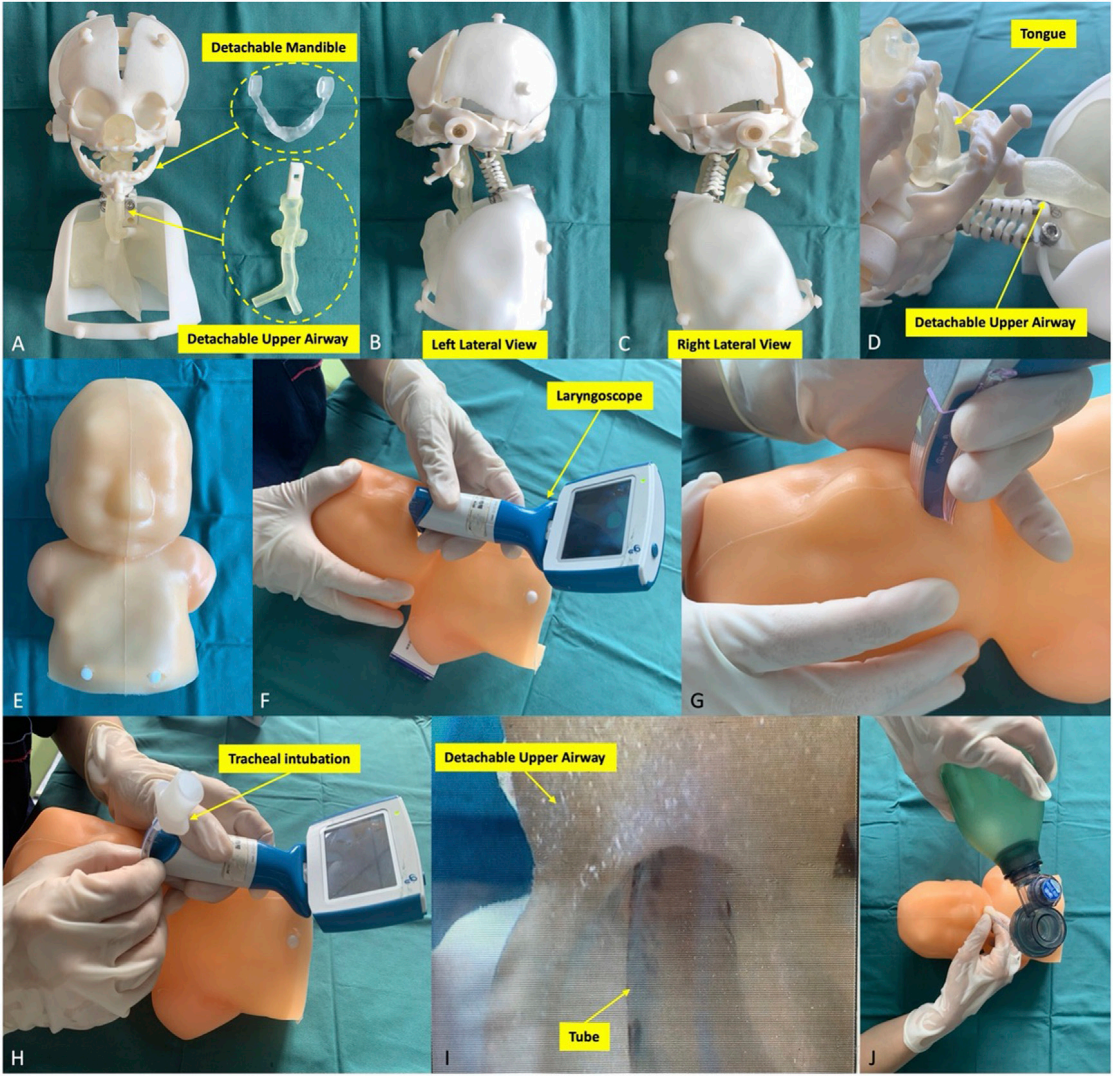
Pierre Robin sequence simulator and its simulation procedures. **(A–E)** The simulator components. The simulator consisted of the head, neck and half of the upper body of the specific patient’s model. The mandible and the upper airway were detachable and replaceable. The simulator was used to simulate different types of laryngoscopes **(F, G)** to complete tracheal intubation **(H, I)**. **(J)** Finally, the anaesthesiologist examined the lung function to verify the simulation effect.

The Digital Imaging and Communications in Medicine (DICOM) format of CT data of PRS patients was imported into Materialise Mimics Version 21.0 software (Leuven, Belgium), and the 3D reconstructed model of the head and airway using the threshold segmentation function was obtained. In Materialise 3-Matic software (Leuven, Belgium), the obtained 3D model was extracted, clipped, smoothed and repaired, and the above anatomical structures were completely restored 1:1. Furthermore, the Standard Triangle Language (STL) file of the 3D reconstructed model was exported to a Stratasys Polyjet 850 multimaterial full colour 3D printer (Supplementary Figure S1, Supplementary Table S1). Different tissues matched hard and soft materials with different colours for editing and printing to obtain a specific PRS simulator (Supplementary Table S1, Figures 2A–E).

### Training and simulation

Twelve anaesthesiologists participated in the training performed 5 times each on the PRS Training Simulator, recording the time of each training (from the beginning use of the laryngoscope to the observance of thoracic undulating) (Figures 2F–J). After the training, they were randomly divided into three groups with a total of 12 nontrained anaesthesiologists, and the simulation was completed on PRS Testing Simulator-2, 3, 4. The training time was recorded, and the performance was evaluated by three chief anaesthesiologists (10-point scale). After the evaluation, all 24 anaesthesiologists filled out the Likert scale scorecard to evaluate the simulator’s performance and function in real-world procedures. The Likert scale contents include the overall perspective, the simulator material and operability, and the performance of simulation steps (Table 1).

**TABLE 1:** Questionnaire for the evaluation of specific training by the Pierre Robin sequence simulator.

Items	Rating				
Overall Perspective					
1. Better understand the anatomical structures and procedural locations	1	2	3	4	5
2. Improve confidence to complete the procedures	1	2	3	4	5
3. Shorten the learning curve	1	2	3	4	5
Simulator Material and Operability					
1. Provide the necessary information about the relevant major pathological findings	1	2	3	4	5
2. Consistency and elasticity similar to that of the human tissue	1	2	3	4	5
3. Authenticity and applicability of the simulation and measurement	1	2	3	4	5
Efficacy and Feasibility of the Simulation					
1. Practice the procedural steps	1	2	3	4	5
2. Skilfully choose the tube diameters	1	2	3	4	5
3. Identify difficulties and improve technical skills	1	2	3	4	5

Note. Ratings are on a Likert scale: 1 (not at all) to 5 (very much).

### Statistical analysis

The measurement data conforming to a normal distribution are presented as the mean ± standard deviation. The rank sum test was used for rating, and the paired Student’s *t-*test was used for analysis. Bilateral *p* values <0.05 were considered statistically significant. All statistical analyses were performed using SPSS software version 26.0 (Armonk, United States).

## Results

### Study population

The demographic characteristics and preoperative CT measurement results of 4 patients with PRS are shown in Table 2. We measured the distance between the inner margins of the mandible (L_1_), the vertical distance from the inner margin of the mandible to L_1_ (L_2_), the distance of the narrowest level of the oropharyngeal region in the upper airway (L_3_), and the distance of the upper airway perpendicular to L_3_ (L_4_). Furthermore, we assessed the cross-sectional area of the glossopharyngeal region of the upper airway (CSA) and the angle between the two inner margins of the mandible and the inner margin of the mandibular body (α). Importantly, the patients’ clinical grades of Pierre Robin sequence were all up to Grade 3 (Lee et al., 2015).

**TABLE 2:** Baseline characteristics of the patients.

Characteristics	Patient #1	Patient #2	Patient #3	Patient #4
Age, days	8	12	41	36
Sex	Male	Female	Male	Male
Height, cm	50	42	48	41
Weight, kg	3.4	2.3	3.0	2.1
Come to term	Yes	Yes	Yes	Yes
Combined with other malformations	Respiratory distress syndrome, Patent ductus arteriosus, Atrial septal defect	Atrial septal defect	Atrial septal defect, Pneumonia	Respiratory distress syndrome, Atrial septal defect
Computed tomography measurements				
L_1_, mm	49.10	42.46	43.76	54.31
L_2_, mm	16.22	16.76	23.42	36.96
L_3_, mm	3.40	3.88	6.59	3.11
L_4_, mm	14.61	15.52	11.81	9.47
CSA, mm^2^	28.06	42.24	68.19	29.63
α, °	113.45	106.23	84.97	72.74
Grading^a^	3	3	3	3
Mallampati classification	IV	IV	IV	IV

L_1_: The distance between the inner margins of the mandible. L_2_: The vertical distance from the inner margin of the mandible to L_1_. L_3_: The distance of the narrowest level of the linguopharyngeal region in the upper airway. L_4_: The distance of the upper airway perpendicular to L_3_. CSA: Cross-sectional area of the glossopharyngeal region of the upper airway. α: The angle between the two inner margins of the mandible and the inner margin of the mandibular body.

^a^ The clinical grading of the Pierre Robin sequence (Cole et al., 2008).

### Baseline characteristics of anaesthesiologists and PRS simulator assembly costs

The baseline characteristics of all 24 anaesthesiologists are shown in Table 3. The average age was 31.8 ± 4.3 years, 66.7% (*n* = 16) were female, and the average number of working years was 6.3 ± 3.1. There were no significant difference among the three groups in terms of average age and working years. Notably, 4 anaesthesiologists in each group were trained on the PRS Training Simulator, and there was no significant difference in the average age or working years between the trained anaesthesiologists and the nontrained anaesthesiologists. As an assembler simulator, the consumables for the detachable part cost 32.76 dollars (of which the mandible model cost 14.08 dollars and the upper airway model cost 18.68 dollars), and other fixed components cost 346.48 dollars (Supplementary Table S1).

**TABLE 3:** Baseline characteristics of the anaesthesiologists.

Group	Number	Sex	Age	Working years	Preprocedural Simulation
Group A	1	Female	34	6	Yes
	2	Male	38	13	Yes
	3	Female	29	5	Yes
	4	Female	26	3	Yes
	5	Female	30	3	No
	6	Female	37	12	No
	7	Male	28	4	No
	8	Female	35	7	No
Group B	1	Male	31	7	Yes
	2	Female	24	2	Yes
	3	Male	29	5	Yes
	4	Female	39	11	Yes
	5	Female	33	6	No
	6	Male	25	3	No
	7	Female	32	6	No
	8	Female	36	10	No
Group C	1	Female	37	9	Yes
	2	Female	34	7	Yes
	3	Female	29	4	Yes
	4	Male	28	3	Yes
	5	Female	33	5	No
	6	Male	31	6	No
	7	Female	27	3	No
	8	Male	38	10	No

### Training, simulation and questionnaire outcomes

**FIGURE 3:**
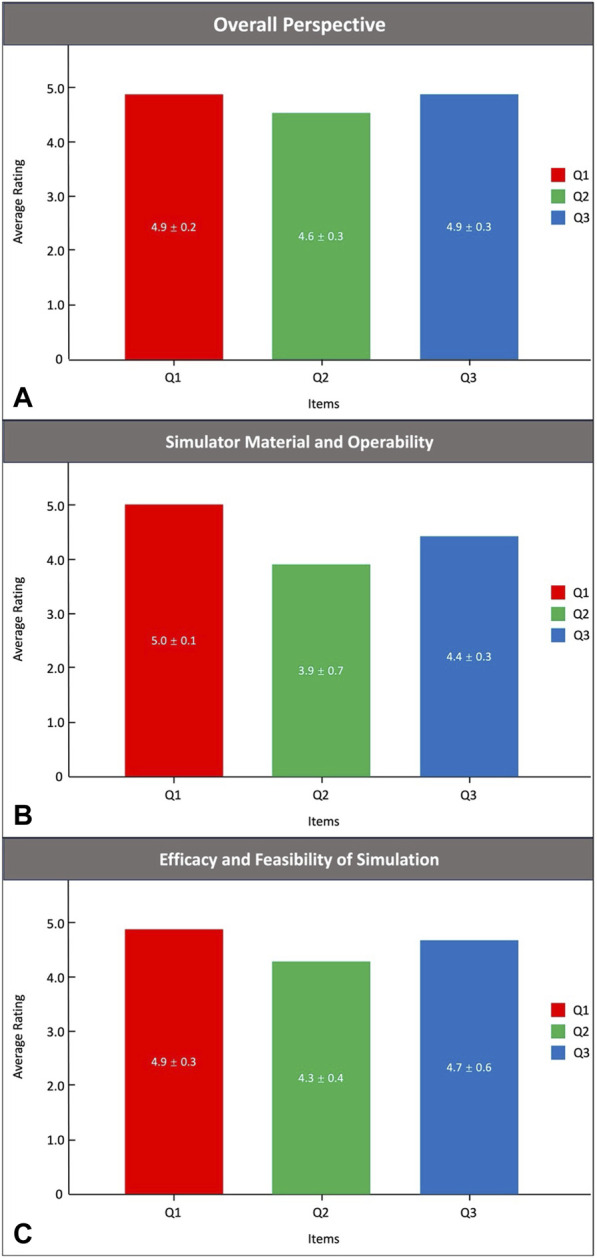
The duration of training on the Pierre Robin Sequence Training Simulator.

**FIGURE 4:**
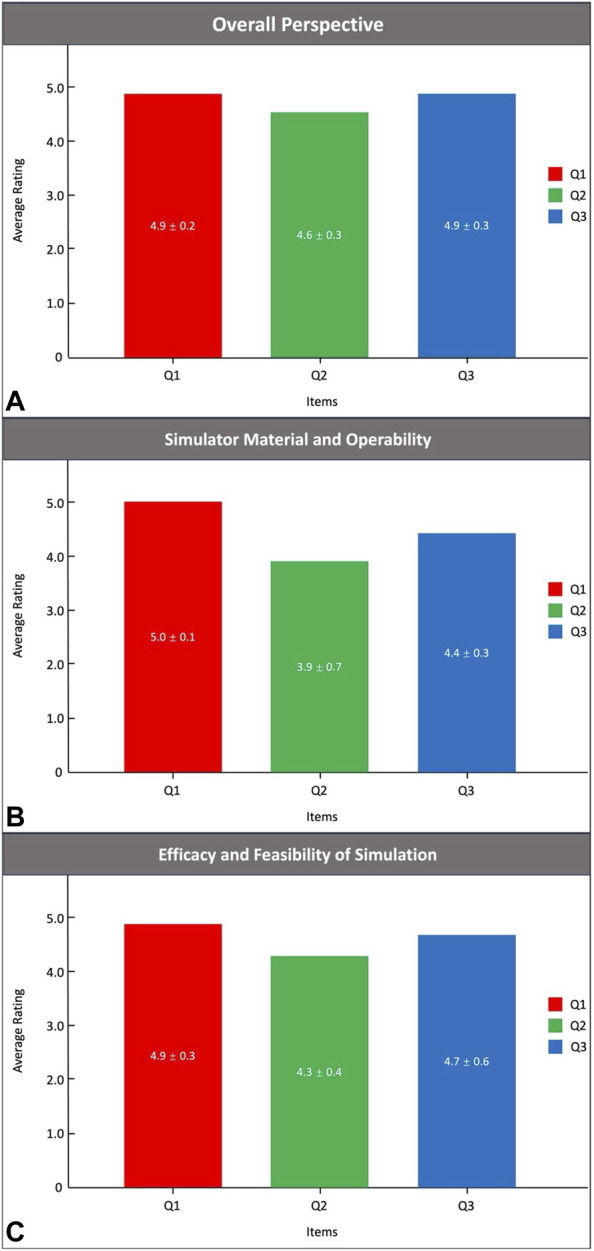
The Likert scale results.

The simulated time results on the PRS Training Simulator are shown in Figure 3. As expected, the time for the 12 anaesthesiologists to complete the training using videoscope gradually decreased (4.3 ± 1.6 min vs. 2.8 ± 1.2 min vs. 2.2 ± 0.9 min vs. 1.4 ± 0.5 min vs. 0.8 ± 0.3 min, *p* < 0.01). The anaesthesiologists of Groups A, B and C completed a simulated operation of TI on PRS Simulator-1, 2 and 3, respectively. Table 4 shows the results of the simulation time. Compared with the nontrained anaesthesiologists, the trained anaesthesiologists had a significantly reduced simulation time (1.2 ± 0.7 vs. 4.3 ± 2.1, *p* < 0.01). Importantly, the simulated performance of the trained anaesthesiologists was significantly better than that of the nontrained anaesthesiologists (8.6 ± 2.2 vs. 5.4 ± 2.0, *p* < 0.01). Notably, all 24 anaesthesiologists approved the reliability and efficacy of the PRS simulator (Figure 4).

**TABLE 4:** Simulation performance among the three groups.

Performance	Simulation	Non-simulation	*p*-value
Simulation Time, min			
Group A	1.3 (0.7, 1.8)	4.5 (2.8, 5.4)	<0.001
Group B	1.0 (0.4, 1.5)	3.8 (2.3, 6.0)	<0.001
Group C	1.2 (0.5, 2.0)	4.6 (2.6, 8.3)	<0.001
Expert Evaluation Rating			
Group A	8.5 (7.0, 10.0)	5.5 (4.0, 8.0)	0.024
Group B	9.0 (8.0, 10.0)	5.8 (5.0, 7.0)	0.017
Group C	8.3 (8.0, 9.0)	5.0 (4.0, 7.0)	0.014

Values are expressed as medians and were analysed by the paired Student’s *t-*test.

## Discussion

This study presents an optimised TI simulator for anaesthesiologists. The results show that the PRS simulator can help significantly shorten the successful TI time defined by beginning of laryngoscope and spotting of chest undulating. The trained group was able to cut average time from 4.3 min at first attempt to 0.8 min at 5^th^ one. When facing a different PRS Train Simulator, their successful TI time was also significantly shorter compared to the groups of not being simulated.

The main clinical features of PRS are micrognathia, glossoptosis, and airway obstruction (Robin, 1923), and approximately 58%–90% of patients have a cleft palate (Maas and Poets, 2014). The aetiology of PRS is not completely clear, but it may be related to inclusion body virus infection of giant cells in the uterus (Jakobsen et al., 2007). Previous studies reported that the pathogenesis of PRS may be based on mandibular dysplasia and bilateral symmetric retraction, resulting in a small oral capacity, an abnormal tongue position and secondary damage of palate closure. All those features plus rare occurrence of PRS make their airway handling challenging, a lot of anesthesiologists are not comfortable handing their airways due to lack of training or exposure. Commercially available mannequin are expensive, not readily available and provide only one dimension of airway difficulty (Parotto et al., 2017; Park et al., 2021). In our study, due to the anaesthesiologists could practice various 3D printed models of PRS, and the procedural plan may be formulated by using the simulator to improve the success rate of TI and reduce the potential complications from repeated airway instrumentation.

Difficult airways are one of the most urgent clinical challenges in anaesthesia, and it is very important to fully understand and prepare them before surgery. Based on the accurate design, 3D printing can be used to create highly simulated models, which is one of the essential factors that improve the quality of simulations and reduce the learning curve of anaesthesiologists. First, the accurate estimation of 3D spatial structures are the unique advantages of 3D printing. Anaesthesiologists often face problems with 3D anatomical structures, and 3D printing has shown its important role in dealing with these challenges (Gauger et al., 2018). In a previous study of cricothyroid incisions, 3D reconstruction may highly restore real anatomical structures and distinguish thyroid cartilage, cricoid cartilage and trachea (Gauger et al., 2018). Similarly, the evaluation of the chief anaesthesiologists in this study also endorsed the fidelity of the PRS simulator. The detachable part of the PRS simulator cost 32.76 dollars, and it was able to be used not only to train anaesthesiologists to grasp procedural skills quickly but also to simulate specific challenging cases before procedures.

Furthermore, 3D-printed models may not only be employed to visually identify anatomical structures but also help anaesthesiologists develop preprocedural airway management plans to reduce the incidence of intubation complications. Bustamante et al. (2014) and Parotto et al. (2017) produced a simulated trachea-bronchial tree model with 3D printing for the clinical training of residents to promote their proficiency in trachea-bronchial anatomy, fibreoptic bronchoscopy operation and lung isolation. Han et al. (2016) reported a case of 3D printing used for airway evaluation. A preoperative CT scan of this patient revealed a soft tissue mass at the tracheotomy position. The anaesthesiologist used 3D printing to develop a simulated airway and found scar contraction around the tracheal incision and slight narrowing below the incision. With the guidance of the model, they successfully achieved airway control. In addition, Park et al. (2021) reported a case of using a 3D printed model to simulate TI before anaesthesia to improve the quality of airway management. Meanwhile, Shaylor et al. (2023) carried out a randomized trial of the video laryngoscope to the flexible fiberoptic bronchoscope in a Pierre Robin manikin, the first-attempt success rates were 88.3% and 85.0%, respectively. In this study, the trained anaesthesiologists made significant improvements in simulation performance after training, and the training time on the training simulator was gradually reduced. Importantly, the training performance of the trained anaesthesiologists was also significantly better than that of their nontrained counterparts.

There are some limitations in this study. First, the sample size of this study is limited. We conducted the study and analysis on the models of only 4 PRS patients, and more patients should be included for evaluation in the future. Second, none of the anaesthesiologists performed TI on real PRS patients to further confirm the reliability of the simulator. Third, because the manufacturing of 3D-printed models is relatively time-consuming (9.5 days of average production time were used in the study), which needs to be reduced in the next step of simulator development. Finally, the physical properties of each anatomical structure of the simulator in terms of elongation at break of and tensile strength of various tissues are still different from those of the real human body, which needs to be improved in the next step.

## Conclusion

In this study, a new potential training tool to optimise teaching and training for PRS cases was proposed. The simulator can help anaesthesiologists significantly shortening successful TI time without causing any harm.to real patients. The anaesthesiologists considered our PRS simulator is of high value in application and training. The performance of the trained anaesthesiologists was not only improved after training but also significantly better than that of nontrained anaesthesiologists. Although the simulator is not yet able to give anaesthesiologists a completely realistic simulation experience, it may greatly improve the success rate and efficacy of TI in PRS patients. Therefore, the reliable simulation quality of the simulator makes it promising for clinical applications in the future, and future studies will investigate the effects of using this simulator as a training tool and the potential impact on difficult airway management.

## Data Availability

The original contributions presented in the study are included in the article/Supplementary Material, further inquiries can be directed to the corresponding authors.

## Abbreviations

CT, computed tomography; CSA, cross-sectional area; MDO, mandibular distraction osteogenesis; PRS, Pierre Robin sequence; 3D, 3-dimensional.
