# Corrigendum: Tracheal intubation in patients with Pierre Robin sequence: development, application, and clinical value based on a 3-dimensional printed simulator

**DOI:** 10.3389/fphys.2024.1400492

**Published:** 2024-06-17

**Authors:** Yu Mao, Lu Liu, John Zhong, Pei Qin, Rui Ma, Mingzhang Zuo, Li Zhang, Lifang Yang

**Affiliations:** ^1^ Department of Cardiovascular Surgery, Xijing Hospital, Air Force Medical University, Xi’an, China; ^2^ Department of Anesthesiology, Children’s Hospital of Nanjing Medical University, Nanjing, China; ^3^ Department of Anesthesiology and Pain Management, University of Texas Southwestern Medical Center, Dallas, TX, United States; ^4^ Department of Anesthesiology, Xi’an Children Hospital, Xi’an, China; ^5^ Department of Anesthesia, Beijing Hospital, National Center of Gerontology, Institute of Geriatric Medicine, Chinese Academy of Medical Sciences, Beijing, China

**Keywords:** Pierre Robin sequence, tracheal intubation, 3-dimensional printing, simulator, difficult airway

In the published article, there was an error in **Affiliation 2**. Instead of “Department of Anesthesiology, Nanjing Children Hospital, Nanjing, China”, it should be “Department of Anesthesiology, Children's Hospital of Nanjing Medical University, Nanjing, China.”

In the published article, there was an error regarding the affiliation for Pei Qin and Rui Ma. Instead of “Department of Anesthesiology, Nanjing Children Hospital, Nanjing, China”, it should be “Department of Anesthesiology, Xi'an Children Hospital, Xi'an, China”.

In the published article, there was an error in **Correspondence**. Instead of “Li Zhang, drzhangli@njmuedu.cn”, it should be “Li Zhang, drzhangli@njmu.edu.cn.”

The authors apologize for these errors and state that this does not change the scientific conclusions of the article in any way. The original article has been updated.

