# Supplementary material for: Tracheal intubation in patients with Pierre Robin sequence: development, application, and clinical value based on a 3-dimensional printed simulator
Source: Front Physiol. 2024 Feb 1;14:1292523. doi: 10.3389/fphys.2023.1292523 (PMC10875733; doi:10.3389/fphys.2023.1292523)
Supplement: Supplementary file 1 [file Table1.docx]

**Supplemental Material**

**Table S1.** Comparison of Mechanical Properties Between Human and 3-Dimensional Printed Model Material and Printing Costs for Each Patient

| **Tissue** | **Human**^1-3^ | **3-Dimensional Printed Model^*^** |
| --- | --- | --- |
| Tensile strength of skull | 5500 MPa | 3100 MPa |
| Elongation at break of skull | 300% | 240% |
| Tensile strength of mandible | 1600 MPa | 2400 MPa |
| Elongation at break of mandible | 300% | 220% |
| Tensile strength of tongue | 1400 KPa | 2.4 - 3.1 MPa |
| Elongation at break of tongue | 500% | 220 - 240% |
| Tensile strength of upper airway | 7.54 KPa | 2.4 - 3.1 MPa |
| Elongation at break of upper airway | 450% | 220 - 240% |
| Tensile strength of skin | 600 KPa | 10 – 20 MPa |
| Elongation at break of skin | 400% | 550 – 650% |
| **Items** | **Mean Time Cost** | |
| Reconstruction time for each 3-dimensional printed model (Undetachable parts) | 1.25 days | |
| Printing time for each 3-dimensional printed model (Undetachable parts) | 5.50 days | |
| Reconstruction time for each 3-dimensional printed model (Detachable parts) | 0.75 days | |
| Printing time for each 3-dimensional printed model (Detachable parts) | 2.00 days | |
| **Items** | **Mean Expense Cost** | |
| Printing expenditure for each 3-dimensional printed model (Skull) | $223.81 | |
| Printing expenditure for each 3-dimensional printed model (Mandible) | $14.08 | |
| Printing expenditure for each 3-dimensional printed model (Tongue) | $7.32 | |
| Printing expenditure for each 3-dimensional printed model (Upper Airway) | $11.36 | |
| Printing expenditure for each 3-dimensional printed model (Skin) | $122.67 | |

^*^All the 3-dimensional printed models used in this study were printed using the Stratasys Polyjet 850 multimaterial full-color 3-dimensional printer (Stratasys Ltd, Eden Prairie, MN, USA).

The material comprising the skull is Agilus30 (FLX985, FLX935).

The material comprising the mandible is Agilus30 (FLX985, FLX935).

The material comprising the tongue is Agilus30 (FLX985, FLX935).

The material comprising the upper airway is Agilus30 (FLX985, FLX935).

The material comprising skin is K-9000.


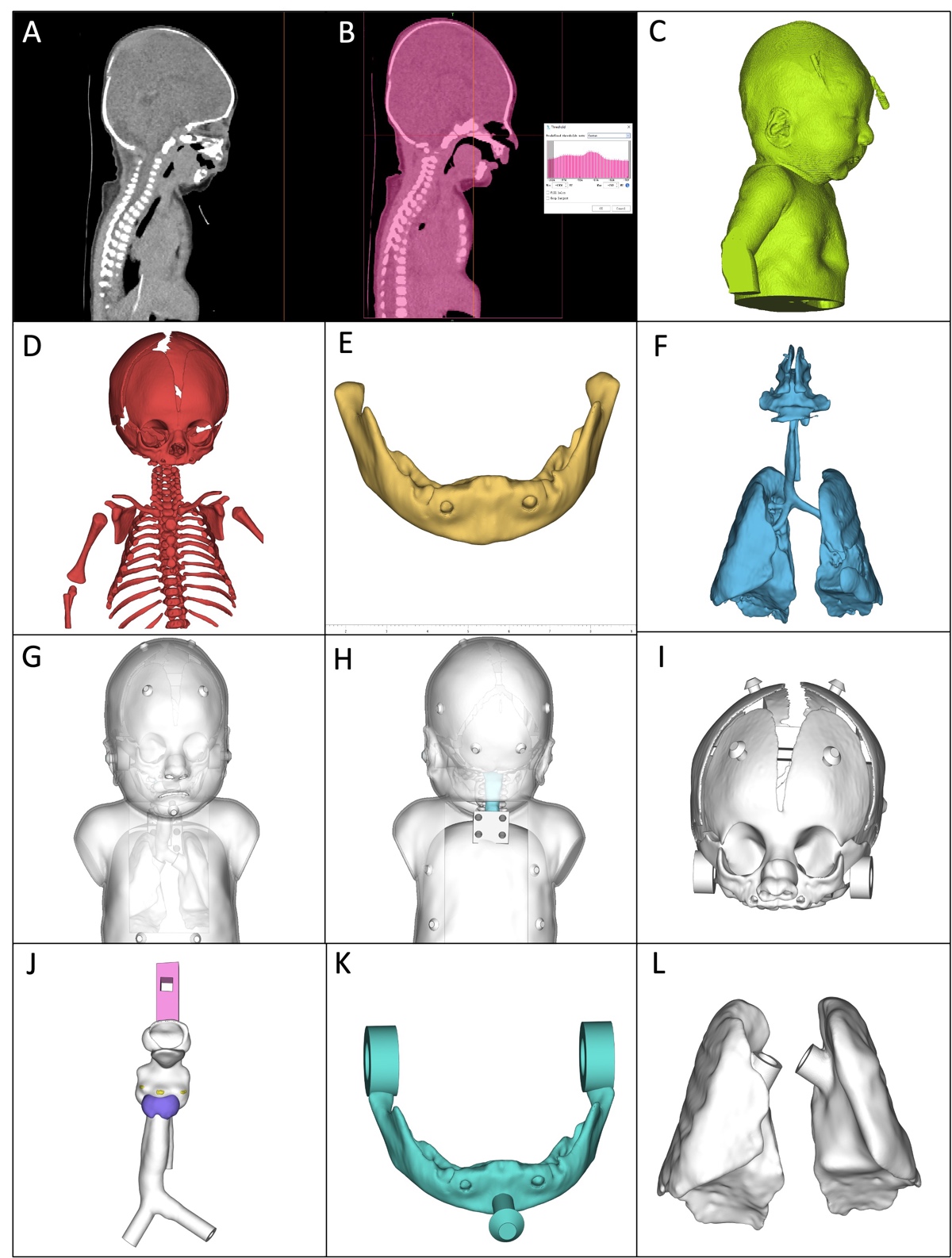


**Figure S1.** The 3-dimensional reconstruction process of the Pierre Robin sequence simulator. (A) The original computed tomography scan. (B) Threshold segmentation. (C) Extraction. (D-F) The reconstructed model of the skull, mandible, upper airway and lungs was clipped and smoothed. (G, H) The assembled reconstructed model was completed, and the front view and the back view are displayed. (I-L) The reconstructed model of the skull, detachable part (including the mandible, tongue and upper airway), mandible and lungs.

**References**

1. Zhao M, Barber T, Cistulli PA, et al. Simulation of upper airway occlusion without and with mandibular advancement in obstructive sleep apnea using fluid-structure interaction. J Biomech 2013; 46(15): 2586-2592.

2. Zhu JH, Lee HP, Lim KM, et al. Passive movement of human soft palate during respiration: A simulation of 3D fluid/structure interaction. J Biomech 2012; 45(11): 1992-2000.

3. Faverani LP, Barao VA, Ramalho-Ferreira G, et al. The influence of bone quality on the biomechanical behavior of full-arch implant-supported fixed prostheses. Mater Sci Eng C Mater Biol Appl 2014; 37: 164170.
